# Brain Phenotype of Transgenic Mice Overexpressing Cystathionine β-Synthase

**DOI:** 10.1371/journal.pone.0029056

**Published:** 2012-01-12

**Authors:** Vinciane Régnier, Jean-Marie Billard, Sapna Gupta, Brigitte Potier, Stéphanie Woerner, Evelyne Paly, Aurélie Ledru, Sabrina David, Sabrina Luilier, Jean-Charles Bizot, Guido Vacano, Jan P. Kraus, David Patterson, Warren D. Kruger, Jean M. Delabar, Jaqueline London

**Affiliations:** 1 Unité de Biologie Fonctionnelle et Adaptative, CNRS EAC 4413, Université Paris Diderot, Sorbonne Paris Cité, Paris, France; 2 Centre de Psychiatrie et Neurosciences, INSERM UMR 894, Université Paris Descartes, Sorbonne Paris Cité, Paris, France; 3 Cancer Biology Program, Fox Chase Cancer Center, Philadelphia, Pennsylvania, United States of America; 4 Key-Obs SAS, Orléans, France; 5 The Eleanor Roosevelt Institute and the Department of Biological Sciences, University of Denver, Denver, Colorado, United States of America; 6 Department of Pediatrics, University of Colorado School of Medicine, Aurora, Colorado, United States of America; Consejo Superior de Investigaciones Cientificas, Spain

## Abstract

**Background:**

The cystathionine β-synthase (*CBS*) gene, located on human chromosome 21q22.3, is a good candidate for playing a role in the Down Syndrome (DS) cognitive profile: it is overexpressed in the brain of individuals with DS, and it encodes a key enzyme of sulfur-containing amino acid (SAA) metabolism, a pathway important for several brain physiological processes.

**Methodology/Principal Findings:**

Here, we have studied the neural consequences of CBS overexpression in a transgenic mouse line (60.4P102D1) expressing the human *CBS* gene under the control of its endogenous regulatory regions. These mice displayed a ∼2-fold increase in total CBS proteins in different brain areas and a ∼1.3-fold increase in CBS activity in the cerebellum and the hippocampus. No major disturbance of SAA metabolism was observed, and the transgenic mice showed normal behavior in the rotarod and passive avoidance tests. However, we found that hippocampal synaptic plasticity is facilitated in the 60.4P102D1 line.

**Conclusion/Significance:**

We demonstrate that CBS overexpression has functional consequences on hippocampal neuronal networks. These results shed new light on the function of the *CBS* gene, and raise the interesting possibility that CBS overexpression might have an advantageous effect on some cognitive functions in DS.

## Introduction

Down syndrome (DS) is a genomic disorder, caused by total or partial trisomy of human chromosome 21 (Hsa21), which occurs in about 1/800 live births [1]. The clinical presentation is complex, with more than 80 clinical features being described [2]. Penetrance and expressivity of DS phenotypic traits are highly variable in affected people. Intellectual disability (ID) is one of the few phenotypes with full penetrance, although its intensity can vary from severe to moderate (IQ = 25–55) [3]. Neuropsychological examination of persons with DS has indicated particular impairment in motor skills, language, verbal short term memory and in explicit long-term memory, a hippocampally mediated memory [4], [5].

Although the main genetic aetiology of DS is known, i.e. an extra copy of some Hsa21 material, much remains to be learnt about the molecular processes involved in the pathogenesis of the disorder. More than 400 genes have been assigned to Hsa21 (NCBI genome build 37.1). High throughput gene expression studies have demonstrated an increase in Hsa21 gene expression as a primary gene dosage effect [6], [7], [8], [9], [10], but the analysis of samples with high statistical power has also indicated that only a limited number of Hsa21 genes are significantly overexpressed, therefore suggesting a prominent role of those dosage-sensitive genes in the DS phenotype [6], [10].

In order to understand which dosage-sensitive genes contribute to the DS cognitive defects, mouse model approaches have been used. Hsa21 is syntenic to genomic regions located on mouse chromosomes 10, 16 and 17 (Mmu10, Mmu16 and Mmu17) (www.ensembl.org). Mice with segmental trisomy of those chromosome regions were generated [11], [12], [13], [14], [15], [16], which culminated in a mouse model bearing triplicated regions for all human chromosome 21 syntenic regions [17]. Behavioral and neurophysiological characterization of those models pointed to the involvement of the Mmu16 and Mmu17 regions orthologous to Hsa21 in cognitive function (see [18], [19] for review). However, complex genotype-phenotype correlations were revealed, suggesting that the neurobiological phenotype results from the interaction of several genes or regions which can have either negative or positive effects on learning and memory and behavior. As a complementary approach, it is thus important to investigate the contribution of individual genes.

**Figure 1:**
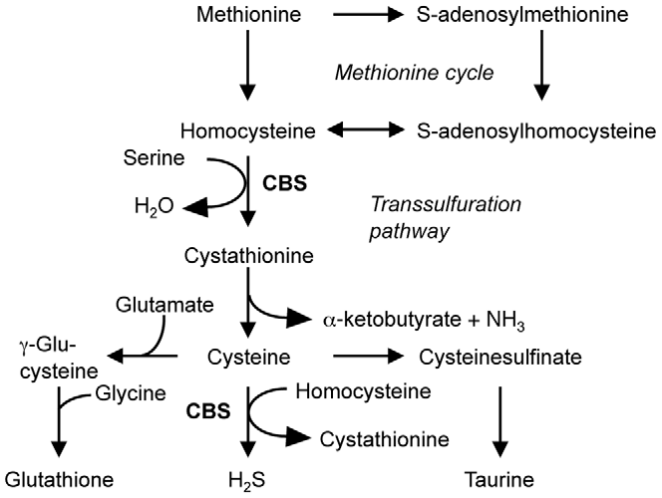
Involvement of CBS in sulfur-containing amino acid metabolism. From Stipanuk [54].

The Hsa21 *CBS* gene coding for the cystathionine β-synthase enzyme (EC 4.2.1.22), which has a mouse orthologue on Mmu17, is likely to be relevant to the DS cognitive profile. It belongs to the family of Hsa21 genes that are overexpressed in the brain of DS patients, with a 1.8-fold increase at the mRNA level in dorsolateral prefrontal cortex [8], and a ∼2.3–2.9-fold increase at the protein level in frontal lobes [20]. CBS is involved in sulfur-containing amino acid (SAA) metabolism (fig. 1), catalysing the the first step in the transsulfuration pathway, where the β-replacement of L-serine with L-homocysteine leads to the formation of cystathionine and water [21]. CBS can catalyse an alternative β-replacement reaction, where cysteine is used in place of serine, resulting in the formation of cystathionine and H_2_S [22]. Imbalance in SAA metabolism has been reported in individuals with DS [23], [24], [25].

CBS has initially been characterised as a hepatic enzyme [26], [27], but clues have accumulated indicating a role in brain function. CBS protein is present in all cerebral regions [28], [29], and in mouse, it has been shown that *Cbs* gene expression is highly regulated during the development of the central nervous system [29], [30]. The transsulfuration pathway diverts homocysteine from the methionine cycle, and leads to cysteine formation. Albeit initially subject to controversy, the existence of a functional transsulfuration pathway in brain has now been established [31]. Moreover, several metabolite compounds from SAA metabolism play important brain cellular functions: S-adenosylmethionine (SAM) is a methyl donor, involved in neurotransmitter synthesis [32]; glutathione and taurine are cellular redox-controlling molecules [33], [34], and H_2_S plays a role in synaptic plasticity and neuroprotection [35].

A first transgenic mouse model containing the human *CBS* cDNA under control of the zinc-inducible metallothionein promoter (MT-I) has been created [36]. This model did express the human CBS protein, but not in the brain, due to the tissue specificity of the MT-I promoter. Three other transgenic lines have been produced (KB2007G4, P102D1, 60.4P102D1), bearing different human genomic fragments encompassing the *CBS* gene [37]. Initial RT-PCR analysis experiments have shown expression of the human *CBS* mRNA in the brain of these transgenic mice [37]. The 60.4P102D1 line, which contains only the *CBS* gene, is potentially useful for the analysis of the consequences of the function of an extra copy of *CBS* in the absence of expression of other human transgenes. However, previous work did not assess the quantitative levels of CBS expression nor whether and to what extent total CBS activity was altered in these mice. Here, we have further characterized the 60.4P102D1 line. We showed that this line of transgenic mice indeed expresses human CBS protein in the brain, and performed quantitative analysis of CBS expression and activity in different brain areas of the transgenic mice. We also determined the consequences of CBS overexpression on brain amino acid metabolism as well as on hippocampal synaptic plasticity. We finally tested the transgenic mice for two behavioral tasks related to cerebellar and hippocampal functions.

## Results

### Transgene copy number in the *CBS* 60.4P102D1 transgenic line

FISH analysis of the 60.4P102D1 transgenic line showed a single insertion site, on a chromosome different from the mouse chromosome 17, the location of the endogenous mouse *Cbs* gene [37]. However, the number of copies of the *CBS* gene that integrated at that site was not determined. We therefore determined the number of *in situ* copies of the human *CBS* transgene in hemizygous transgenic mice (referred to here as Tg*hCBS*60.4 mice) by quantitative PCR using human DNA as a calibrator. Human specific *CBS* primers were used for detection of total copy number, and primers for both human *SIM2* and mouse *Sim2* for normalization. The fold change in copy number of the *CBS* gene in the hemizygous transgenic samples relative to the human sample used as a calibrator was evaluated by the 2^−ΔΔCt^ method [38]. We found a mean value of 1.02±0.03, indicating that 2 copies of the *CBS*-bearing human genomic fragment were integrated in the 60.4P102D1 line.

### CBS protein expression profile in Tg*hCBS*60.4 and control mice

**Figure 2:**
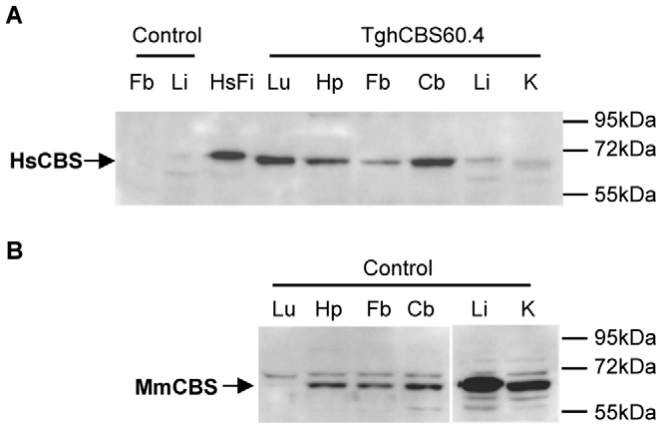
Western blot analysis of CBS expression in Tg*hCBS*60.4 and control mouse tissues. Lu = Lung, Hp = Hippocampus, Fb = Forebrain, Cb = Cerebellum, Li = Liver, K = Kidney. (A) The human 63-kDa CBS protein (HsCBS) was detected using the anti-NH_2_hCBS polyclonal antibody. Human specificity of the antibody was assessed by the absence of immunoreactivity with control mouse tissues (liver and forebrain) and immunoreactivity with cultured human fibroblasts (HsFi). (B) The mouse 63-kDa CBS protein (MmCBS) was detected using the anti-hCBS polyclonal antibody. Coomassie blue staining was used as a loading control (Figure S1).

The human *CBS* gene encodes a 551 amino-acid protein that shares 84% identity with the 547 amino-acids encoded by its murine counterpart. We used an anti-human CBS specific antibody (anti-NH_2_hCBS) to examine the production of a translated protein from the transgene in various Tg*hCBS*60.4 mouse tissues. We detected the expected 63-kDa polypeptide [39] in the brain and lung of transgenic animals but found almost no immunoreactivity in liver or kidney extracts (fig. 2A). As this polypeptide migrated slightly faster than the human protein detected in cultured human fibroblasts, we assessed the integrity of the *CBS* transgene coding sequence. Sequencing of human specific RT-PCR products from Tg*hCBS*60.4 brain tissue showed that the cDNA produced from the transgene encoded the predicted 551 amino acid human CBS protein (data not shown).

We next compared the expression profile of the human protein with the expression profile of the endogenous protein in control mouse tissues. Immunodetection of the mouse CBS was performed with an antibody that can recognize both the human and mouse CBS protein (anti-hCBS) (fig. 2B). The immunoblot showed that endogenous CBS was highly expressed in liver and kidney, moderately in brain, and not detected in lung, as previously described [40]. In brain, it was most highly expressed in cerebellum, followed by hippocampus, and lastly by forebrain. The same brain regional profile was observed for the human protein in Tg*hCBS*60.4 mice (fig. 2A).

### Increase in CBS expression and activity in different brain regions of Tg*hCBS*60.4 mice

**Figure 3:**
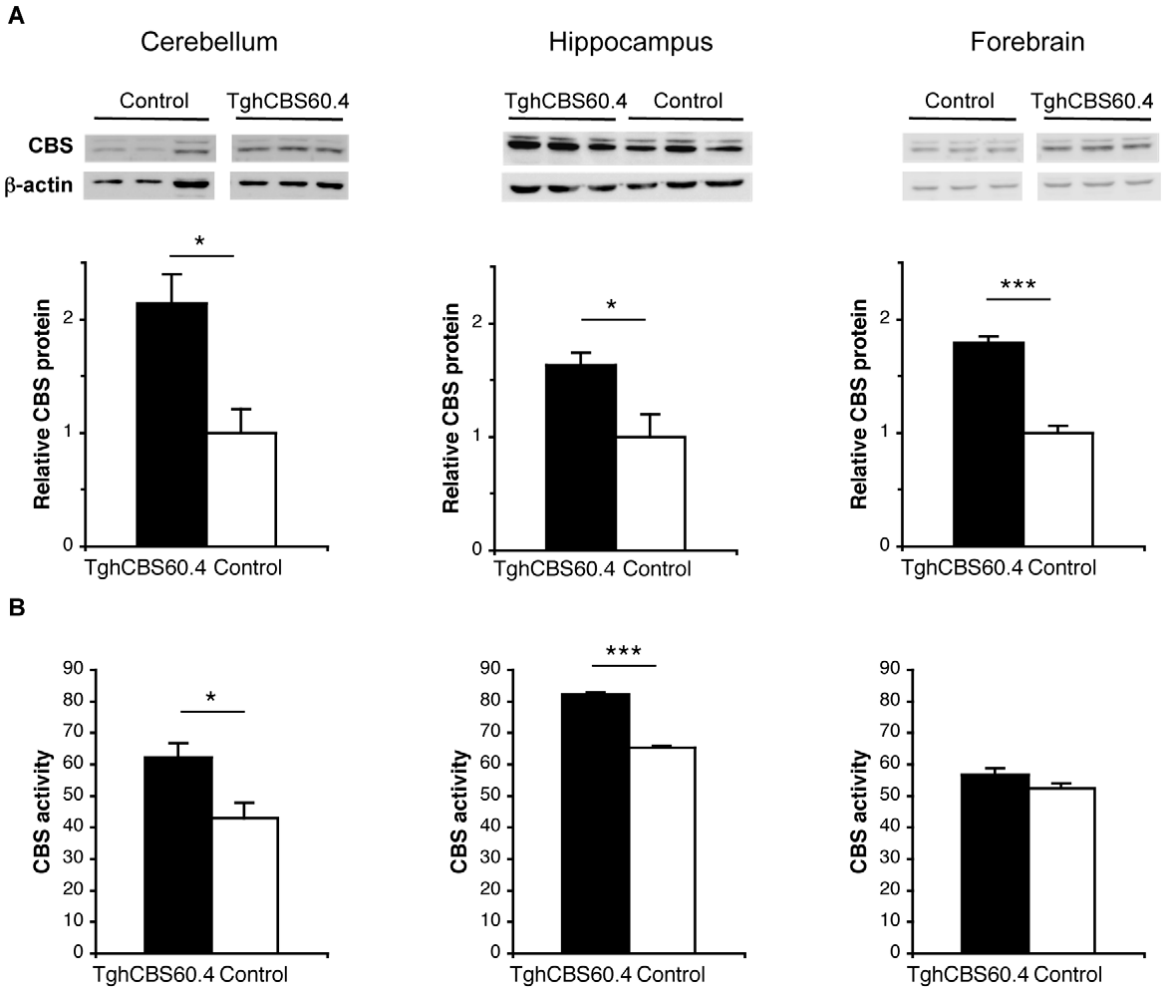
CBS expression and activity in brain regions of Tg*hCBS*60.4 mice. (A, top) Immunoblots for CBS and β-actin in cerebellum (left panel), hippocampus (middle panel) and forebrain (right panel). Total CBS proteins (human CBS plus mouse CBS) were detected using the anti-hCBS polyclonal antibody that recognises both the human and mouse proteins. (A, bottom) Quantification of CBS proteins normalized to β-actin and relative to control mice in the three brain regions (*n* = 3 Tg*hCBS*60.4 and *n* = 3 control) (B) CBS activity in cerebellum (left panel), hippocampus (middle panel) and forebrain (right panel). Units are nanomoles of cystathionine formed per milligram of protein extract per hour. For cerebellum and forebrain, data represent mean ± S.E.M. of activity measurements in each group (*n* = 9 Tg*hCBS*60.4 and *n* = 7 control; *n* = 8 Tg*hCBS*60.4 and *n* = 8 control, respectively). For hippocampus, data represent mean ± S.E.M. of three experimental assays performed on same pooled hippocampi (*n* = 7 Tg*hCBS*60.4 and *n* = 8 control). *for *p*<0.05. ***for *p*<0.001.

We next compared the total CBS (mouse CBS plus human CBS) protein level in different brain regions of transgenic and control mice. A significant increase was found in cerebellum (∼2.1-fold), forebrain (∼1.8-fold) and hippocampus (∼1.6-fold) of transgenic mice (fig. 3A).

In order to determine if CBS overexpression in the Tg*hCBS*60.4 mice resulted in an increase in enzyme activity in the different brain regions, we measured CBS activity in extracts using the standard β-replacement reaction assay, i.e. the formation of cystathionine from serine and homocysteine. In cerebellum and hippocampus of transgenic mice (fig. 3B, left and middle panel), we found that the mean activity was significantly enhanced compared to controls (62.3±4.5 nmoles cystathionine/mg protein/hr *vs*. 43.0±5.0 nmoles cystathionine/mg protein/hr and 82.2±0.5 nmoles cystathionine/mg protein/hr *vs*. 65.3±0.06 nmoles cystathionine/mg protein/hr, respectively). CBS overexpression therefore resulted in a ∼1.3–1.4-fold increase in CBS activity in those brain regions. Surprisingly, no increase in activity was found in the forebrain of transgenic mice (56.8±2.0 nmoles cystathionine/mg protein/hr *vs*. 52.5±1.5 nmoles cystathionine/mg protein/hr for control mice; fig. 3B, right panel). Altogether, these experiments demonstrate that the 60.4P102D1 mouse line is a valid model in which to assess the functional consequences of CBS overexpression in brain. Therefore, we undertook the analysis of these consequences.

### Metabolic profiling of Tg*hCBS*60.4 mice brain

We next examined the effect of CBS overexpression on sulfur amino-acid metabolism and other metabolic compounds in the brain of transgenic mice. As the highest fold increase in total CBS proteins and CBS activity was observed in the cerebellum of transgenic animals, we used this region to determine the concentration of 30 different amino acids and metabolite compounds in transgenic and control mice. No statistical difference was found for any metabolite compound under analysis and the Tg/Control ratio for each amino acid ranged from 0.89–1.27 (table 1). However, the level of cystathionine, the immediate downstream product of CBS, was slightly increased in transgenic mice, with Tg/Control ratio of 1.27 (*p* = 0.06). The homocysteine concentration was below detectable levels in our analysis. We also examined amino acid levels related to SAA metabolism in the hippocampi of transgenic and control animals, but the small quantities of material required us to pool the hippocampi of each group together for analysis. We found very similar ratios for all the metabolites tested, although the largest difference in ratio (1.18) was again observed for cystathionine (table S1).

**Table 1:** Concentrations (nmoles/mg protein) of 30 amino acids and metabolite compounds in cerebellum extracts of Tg*hCBS*60.4 and control mice.

	Control (n = 7)	Tg*hCBS*60.4 (n = 7)	Ratio Tg/Control	*p*-value
Taurine	151.17±7.17	167.10±4.88	1.11	0.09
Phosphoethanolamine	18.8±0.92	19.23±0.95	1.02	0.75
Thr	11.45±0.73	14.35±1.61	1.25	0.13
Ser	13.72±0.53	15.75±1.11	1.15	0.12
Asn	3.28±0.16	3.49±0.20	1.06	0.44
Glu	220.28±12.5	239.28±8.29	1.09	0.23
Gln	158.08±8.21	162.72±7.66	1.03	0.69
Sarcosine	3.36±0.2	3.34±0.13	0.99	0.92
α-aminoadipic acid	1.2±0.08	1.14±0.07	0.96	0.64
Gly	65.07±3.27	68.38±2.65	1.05	0.45
Ala	20.56±2.4	24.47±2.18	1.19	0.25
Citrulline	0.61±0.05	0.69±0.04	1.15	0.21
Val	2.05±0.08	2.29±0.27	1.12	0.80
Met	1.39±0.10	1.39±0.12	1	0.99
Cystathionine	8.10±0.36	10.27±0.94	1.27	0.06
Ile	0.95±0.22	0.84±0.04	0.89	0.8
Leu	2.02±0.15	2.01±0.08	0.99	0.95
Tyr	1.81±0.15	1.76±0.13	0.97	1
β-Alanine	0.77±0.05	0.79±0.04	1.02	0.78
Phe	1.48±0.08	1.50±0.04	1.01	0.82
γ-aminobutyric acid	58.27±2.19	57.07±1.7	0.98	0.67
Ethanolamine	5.86±0.56	5.87±0.59	1.00	0.38
Ammonia	30.39±2.75	32.95±2.52	1.08	0.51
Ornithine	0.16±0.02	0.19±0.02	1.18	0.25
Lys	3.25±0.24	3.15±0.14	0.97	0.72
His	1.51±0.08	1.57±0.07	1.03	0.63
Carnosine	2.67±0.17	2.41±0.25	0.90	0.4
Arg	2.84±0.18	2.87±0.14	1.01	0.89
Pro	1.5±0.14	1.37±0.07	0.92	0.44
Total cysteine	5.69±0.76	6.3±0.74	1.11	0.57
Total homocysteine	BD	BD	ND	ND

Data correspond to mean ± S.E.M. BD: below detection. ND: not determined.

### Altered long-term synaptic plasticity in Tg*hCBS*60.4 mice

**Figure 4:**
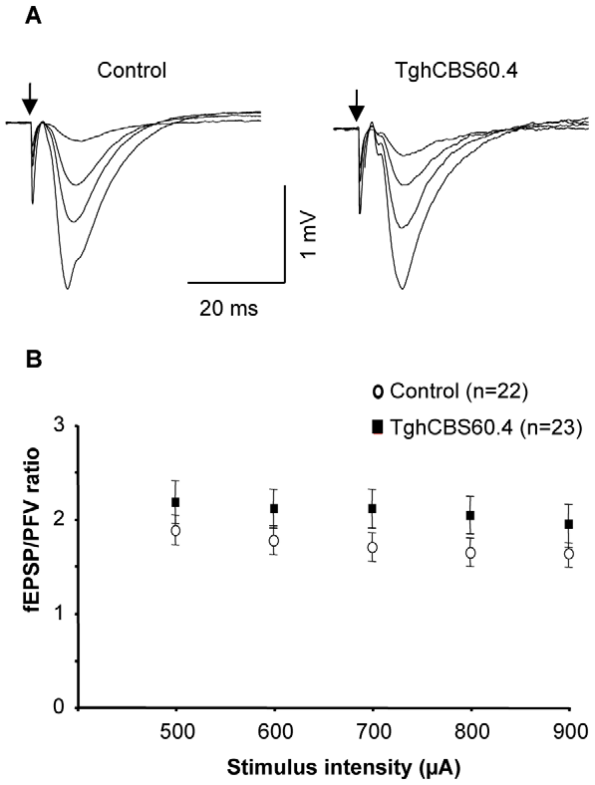
Basal synaptic transmission in Tg*hCBS*60.4 mice. (A) Superimposed sample traces of evoked AMPA-R-mediated fEPSPs induced in a control (left) and a Tg*hCBS*60.4 mouse (right) by increased intensities of electrical stimulation of glutamate afferents (arrow). Traces are averages of 3 consecutive responses. (B) Comparison of synaptic efficacy as determined by the fEPSP/PFV ratio calculated at a stimulus intensity from 500 to 900 µA in Tg*hCBS*60.4 (23 slices/5 animals) and control (22 slices/5 animals) mice.

To determine whether overexpression of CBS has consequences on brain physiology, we performed a series of electrophysiological recordings in hippocampal slices from control and transgenic mice. In the two genotypes, electrical stimulation of glutamatergic afferents in the CA1 *stratum radiatum* induced a presynaptic fiber volley (PFV) followed by a field excitatory postsynaptic potential (fEPSP). This postsynaptic response was blocked at the end of the recording by the antagonist 2,3-Dioxo-6-nitro-1,2,3,4-tetrahydrobenzoquinoxaline-7-sulfonamide (NBQX, 10 µM) indicating the selective activation of AMPA subtype of glutamate receptors. Both responses increased as a function of stimulus intensity (fig. 4A). Comparison of input/output (I/O) curves obtained with current stimulus intensities from 500 to 900 µA showed that the fEPSP/PFV ratio was not significantly different in slices from control and transgenic mice (*F*_4.76_ = 1.06, non-significant), thus indicating that basal synaptic strength was not altered in Tg*hCBS*60.4 mice (fig. 4B).

**Figure 5:**
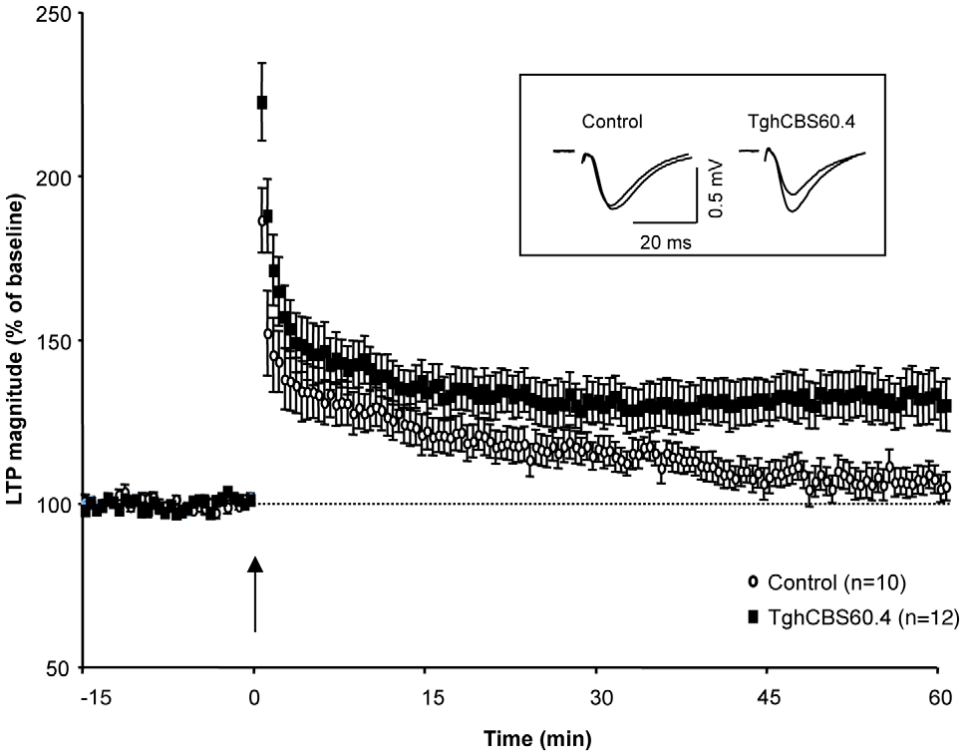
Theta-burst-induced LTP in Tg*hCBS*60.4 mice. Comparison of averaged LTP expressed as percent change in the slope of fEPSP *vs* time, induced by theta-burst stimulation (TBS, arrow) of glutamate afferents and recorded in slices from Tg*hCBS*60.4 (12 slices/9 animals) and control (10 slices/7 animals) mice. In the insert, representative traces of fEPSPs, recorded before and 60 min after TBS, are superimposed.

We then determined whether synaptic plasticity is altered in transgenic mice by evaluating Long Term Potentiation (LTP) using the theta-burst stimulation paradigm (TBS, see Materials and Methods). In slices from control mice, the conditioning stimulation induced a short but not long-term increase in fEPSP slope since multivariate analyses of variance did not reveal significant differences between baseline values and fEPSP slopes recorded from 45 to 60 min after TBS induction (*F*_1.18_ = 2.3, non-significant) (fig. 5). On the contrary, a significant increase in fEPSP slope persisted in slices from transgenic mice at the same delay after the conditioning stimulation (*F*_1.22_ = 19.7, *p*<0.001). As a consequence, the magnitude of LTP measured between 45 to 60 min post-TBS was significantly higher in slices from transgenic mice (132.5%±7.5% of baseline) as compared to slices from control animals (107.5%±4.5%, *F*_1.19_ = 0.5, *p* = 0.02). These data indicate that the expression of the LTP form of synaptic plasticity was facilitated in Tg*hCBS*60.4 mice.

### Behavioral analysis of Tg*hCBS*60.4 mice

**Figure 6:**
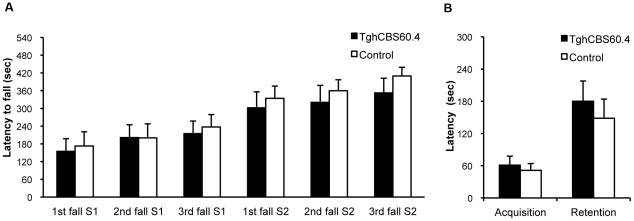
Behavioral assessments in Tg*hCBS*60.4 mice. The analysis was conducted on 8 Tg*hCBS*60.4 and 8 control mice. Data represent mean+S.E.M. for each group. (A) Rotarod test: latencies of first fall, second fall and third fall recorded in the 10 min accelerating periods of the two rotarod sessions (S1, S2) are presented. (B) Passive avoidance test: latency to enter into the dark compartment in acquisition session and in retention session was recorded.

We pursued the characterization of the Tg*hCBS*60.4 mice with some behavioral studies. Visual observation did not reveal any obvious behavioral or morphological differences between Tg*hCBS*60.4 mice and wild-type littermates. We used two paradigms that test a cerebellar-dependent as well as a hippocampal-dependent type of learning. We first assessed motor coordination, balance and motor learning ability with the rotarod test [41], [42], [43]. Performance in this test relies on the integrity of cerebellum and basal ganglia [44], [45]. Transgenic mice did not perform differently from wild type animals either in the first session (difference between groups: *F*_1.14_ = 0.045, *p* = 0.84; interaction group×fall number: *F*_2.28_ = 0.834, *p* = 0.45) or in the second session (difference between groups: *F*_1.14_ = 0.475, *p* = 0.50; interaction group×fall number: *F*_2.28_ = 0.303, *p* = 0.74) (fig. 6A), indicating no alteration in their motor coordination and balance.

Tg*hCBS*60.4 mice were further tested for passive avoidance, a fear-motivated hippocampus-dependent learning task [46], [47], [48]. The latency to enter into the dark compartment ( fig. 6B ) was not significantly different between groups in the acquisition session (*U* = 29.0, *p* = 0.75) and in the retention session (*U* = 26.0, *p* = 0.53). This result shows that the learning skills of the Tg*hCBS*60.4 mice measured by this task were unchanged compared to controls.

## Discussion

In an attempt to understand the contribution of some dosage-sensitive genes in the DS brain, animal models overexpressing individual genes have been created. These models have helped in deciphering the function of those particular genes as well as highlighting the molecular pathways altered after gene imbalance [49]. Most candidate genes studied belong to the HSA21 region orthologous to MMU16 or MMU10. The contribution of individual genes of HSA21 orthologous to MMU17 has been less explored. The cystathionine β-synthase gene is on MMU17. It encodes an enzyme, which lies at a branch point for remethylation and transsulfuration in the sulfur-containing amino acid metabolism. This metabolic pathway furnishes cells with compounds involved in brain function [35], [50], [51]. Butler *et al.* [37] have reported a transgenic mouse line expressing the human *CBS* gene under the control of its own promoter (60.4P102D1). Here, we have performed further phenotypical characterization of this line.

For the 60.4P102D1 mouse line to be a valid model to study the consequences of CBS overexpression in brain, it was necessary to show that transgene expression leads to an effective protein production, and to determine the level of overexpression, since previous studies only demonstrated expression of *CBS* mRNA at an undetermined level [37]. We have shown that 60.4P102D1 mice do produce a human CBS protein in brain, and that the human protein levels vary according to brain regions, with a profile mimicking the endogenous one. The highest expression for the transgenic and endogenous protein was found in cerebellum, in agreement with the mouse CBS brain regional expression analysis previously published [40]. We have also shown that the integrated two copies of the human transgene in hemizygous 60.4P102D1 mice results in the expected ∼2-fold increase in total CBS proteins in the different brain areas of transgenic animals when compared to controls. This increase is near the 2.3–2.9-fold increase in CBS protein levels estimated in the brain of individuals with DS [20]. Taken together, these results indicate that this mouse line meets the criteria that are required to assess functional consequences of CBS overexpression.

We then measured the enzymatic activity resulting from CBS overexpression in the brain of transgenic mice. Interestingly, we found a significant increase in cerebellum and hippocampus, but not in forebrain. The activity was increased by ∼1.3–1.4 times, which is lower than what we found at the protein level. Disruption of a single *Cbs* allele (*Cbs* ^+/−^) also resulted in a change in CBS activity that did not match the amplitude of the change at the protein level [52]. As for the Tg*hCBS*60.4 overexpressing mice, the level of activity in *Cbs* ^+/−^ mice was driven towards the value of controls, while the protein level conformed to the ploidy. These data suggest that post-translational mechanisms of CBS regulation are used in the mouse brain when *Cbs* ploidy is disturbed. Indeed, multiple layers of CBS regulation have been described [53], [54]. Unfortunately, there are no data about CBS activity in the brain of individuals with DS, and it would be interesting to know if such compensatory mechanisms are also present in human brain.

Quantitative analysis of 30 amine-containing metabolic compounds in the cerebellum of transgenic animals did not reveal any major metabolic disturbance due to CBS overexpression, and in particular no main alteration of sulfur-containing amino acid metabolism. We found however, a trend towards a slight increase in cystathionine levels, which is consistent with an increase in flux of homocysteine through the transsulfuration pathway, as expected after elevating CBS activity. Likewise, a limited metabolic profiling of the hippocampus of transgenic animals did not reveal any major disturbance. Lack of any important change in amino acid levels from the methionine or cysteine metabolism pathway has also been observed in the liver of the transgenic mice expressing human *CBS* under the control of the zinc-inducible metallothionein promoter, even though CBS activity in the liver of zinc-treated mice reached 2.2 fold the level of untreated animals [36]. These results suggest a tight control of sulfur-containing amino acid homeostasis in tissues of transgenic animals.

Nevertheless, we observed that CBS overexpression has consequences on functional properties of the hippocampal network. Our electrophysiological data indicate that basal synaptic transmission at schaffer-colateral-CA1 synapses is unaltered in transgenic mice but that the expression of LTP is specifically facilitated. Interestingly, two recently described mouse models carrying either a triplication of the entire Mmu17 region syntenic to Hsa21 (model *Dp(17)1Yey/+*) [16] or triplication of a 12 gene-containing segment from this Mmu17 syntenic region (*Abcg1-U2af1* interval; model Ts1Yah) [12] were also shown to present an increased hippocampal LTP. Both these mouse models are trisomic for *Cbs*. The Ts1Yah mice were checked for expression of *Cbs* along with the other genes trisomic in these animals and the *Cbs* mRNA levels were found to be elevated. CBS protein levels and enzyme activity in the brain of these mice were not assessed. Nonetheless, these authors identified *Cbs* as one of the four genes likely to result in facilitated learning and memory in Ts1Yah mice. Our results, which include demonstration of increased CBS protein and enzyme activity in brain, are the first to show that overexpression of a *CBS* transgene in the absence of other transgenes has functional consequences in the brain, and provide strong argument that CBS is involved in the regulation of synaptic plasticity *in vivo*.

Induction of hippocampal LTP requires the activation of N-methyl-D-aspartate subtype of glutamate receptor (NMDA-R) at post-synaptic cells followed by an influx of calcium ions, which triggers activation of numerous signalling pathways [55]. A challenging question raised by our result is how CBS overexpression can affect this complex mechanism. It has been demonstrated that physiological concentration of H_2_S enhances NMDA-R-mediated responses [56]. One hypothesis could therefore be that CBS overexpression in Tg*hCBS*60.4 mice facilitates LTP by raising the hippocampal hydrogen sulfide concentrations. However, due to the interplay between the transsulfuration pathway and other major cellular metabolic pathways, an alternative hypothesis has to be considered: Indeed, a link between SAM, taurine or glutathione levels and LTP induction has been documented [57], [58], [59]. Further metabolic profiling in the hippocampus of *T*g*hCBS60.4* mice, including SAM, glutathione and hydrogen sulfide concentration measurement, should help in discriminating between the two hypotheses.

LTP is now widely considered as the cellular mechanism underlying learning and memory formation [55], [60], [61]. As the results obtained in the present study indicate the involvement of CBS in this process, we explored possible behavioral changes in the Tg*hCBS*60.4 mice. Unfortunately, we could not assess hippocampal-dependent spatial memory in these mice since the FVB/N background on which they were generated is characterized by retinal degeneration [62], which results in poor spatial awareness [63]. We therefore assayed the mice for contextual learning, which also involves the hippocampus. Tg*hCBS*60.4 mice were subjected to passive avoidance, a contextual-fear learning paradigm. This test did not reveal any difference between the transgenic and control mice. However, avoidance learning also depends on amygdala, entorhinal and parietal cortex [64], therefore we cannot exclude that those structures could counterbalance any change caused by alteration of hippocampal synaptic pathways.

We also investigated whether we could detect any change in sensorimotor learning in the transgenic mice due to the cerebellar overexpression of CBS. The rotarod test showed that CBS overexpression had no consequences on this learning task. This is in agreement with the rotarod result in the Ts1Yah mouse model [12].

In conclusion, we report that the 60.4P102D1 line does overexpress CBS in the cerebellum and hippocampus at levels comparable to what has been estimated in the brain of DS patients and that the overexpression does affect the neurophysiology of the hippocampus, by facilitating LTP. While the link between the hippocampal contextual-learning and LTP remains poorly documented, many studies on transgenic animals have documented that an enhanced LTP in CA1 correlates with better performance in spatial learning, a subtype of explicit memory in which the hippocampus plays a critical role [55], [65]. This raises the interesting possibility that, in the context of the DS phenotype, CBS overexpression does not directly contribute to the spatial long term memory deficits observed in patients, but might, instead, be associated with an advantageous effect on cognitive function.

## Materials and Methods

### Mice

Experiments were carried out in accordance with the European Communities Council Directive (86/609/EEC) regarding the care and use of animals for experimental procedures. This study was approved by the Jacques Monod Institut Animal ethical committee (CEEA-40, approval number CEB-001-2011).

First and second generation hemizygous transgenic 60.4P102D1 mice [37] were generated at the Eleanor Roosevelt Institute Transgenic mouse facility (Denver), and subsequently bred with wild-type FVB/N mice at the Institut Jacques Monod animal facility (Paris). Animals were kept under a 12 h light/dark cycle with unlimited food and water.

At 3–4 weeks of age, descendants were genotyped by PCR analysis of tail DNA using a human-specific *CBS* primers as described in Butler *et al.* [37]. Male mice hemizygous for the human *CBS* transgene (referred to here as Tg*hCBS*60.4 mice) and age-matched controls were compared in each experiment. For experiments performed on tissues, mice were euthanized with CO_2_ and tissues were quickly removed, frozen in liquid nitrogen, and stored at −80°C until used. Brain was dissected on ice to separate cerebellum, hippocampus and forebrain.

### Real-time Quantitative PCR

Transgene copy number in the 60.4P102D1 transgenic line was determined using a method adapted from Ballester *et al.* [66]. Human-specific *CBS* primers (left: 5′AACCTGGGAAGCTGGCATTG3′; right: 5′ATCCCACACACACGCCTGAA3′) and primers common to human *SIM2* and mouse *Sim2* (left: 5′GCTGGACATGTCCCTGTAC3′; right: 5′GCTTCAGGTCAAGGCTGG3′) were designed. The human *SIM2* and mouse *Sim2* were used as reference control genes. Genomic DNA was isolated from the tail of 3 hemizygous mice as well as from a human lymphoblastoid cell line derived from a control individual [6], used as a calibrator. Real time PCR was carried out on genomic DNA using LightCycler 480 SYBR Green I Master kit (Roche Diagnostics) with the above-mentioned primers and LightCycler 480 as recommended by the manufacturers. For each genomic DNA, serial dilutions were prepared (50 ng to 0.048 ng) and each dilution was run in triplicate. A plot of log_10_Input DNA vs ΔCt (Ct*_CBS_*-Ct_control gene_) was done for each DNA sample. Obtention of absolute slopes <0.1 in all cases indicated that PCR efficiencies of *CBS* and control genes were approximately equal in each DNA sample (E*_CBS_* = E_control gene_), thus permitting the use of the 2^−ΔCt^ method (see Applied Biosystems user bulletin #2). The same method was used to assess that PCR efficiencies were the same for each amplicon on mouse or human DNA. The 2^−ΔΔCt^ method was then used to compare the ΔCt value of transgenic animal samples (Ct*_CBS_*-Ct*_Sim2_*) with unknown copy number with the ΔCt of human DNA sample (Ct*_CBS_*-Ct*_SIM2_*). Since transgenic animals were hemizygous for the transgene while the human calibrator was homozygous for *CBS*, the transgene copy number was obtained by multiplying the 2^−ΔΔCt^ value by a factor of two.

### Immunoblotting

Tissues from 4 month-old mice were either directly homogenized (lung, hippocampus, cerebellum, liver) or reduced to powder with mortar and pestle before homogenization (forebrain, kidney) in ice-cold 0.1 M sodium phosphate pH 7.2 (10 µl/mg of tissue) containing protease inhibitor (Complete-Mini, Roche Diagnostics). The homogenate was centrifuged at 12500 g for 10 min at 4°C and the supernatant was retained. Protein concentration was determined using the Bio Rad Bradford protein assay following the manufacturer's instructions. Equal amounts of protein (20 µg) were separated on a 10% SDS-polyacrylamide gel and electrotransferred onto nitrocellulose membranes (Amersham Biosciences). Antibody incubations were done in TBST (50 mM Tris-HCl, pH 8, 150 mM NaCl and 0.5% Tween) containing 4% skimmed milk. Two different affinity-purified polyclonal rabbit anti-human CBS antibodies were used: anti-hCBS (1∶5000; previously described in Kraus [67]), raised against the full length human CBS protein was used for simultaneous detection of human and mouse CBS proteins, and anti-NH_2_hCBS (1∶1000) raised against the synthetic peptide (GCPASESPHHHTAPAK, Biosynthesis, Inc.) was used for specific detection of the human CBS protein. Monoclonal anti-β actin antibody (1∶4000, clone AC-15, Sigma) was used as loading control. Primary antibody was detected with the species-appropriate HRP-conjugated secondary antibody (anti-rabbit IgG, 1∶2000 or anti-mouse IgG, 1∶40000, Sigma). Signal was visualized with the LAS-3000 Image reader (Fujifilm) after treatment with western blotting luminol reagent (Santa Cruz Biotechnology). Densitometric analyses were performed with Science Lab 2003-Image Gauge v4.2 Software (Fujifilm). Each experiment was repeated twice.

### Cystathionine β-synthase (CBS) activity measurement

Brain tissues from 6–7 month-old mice were homogenized in extraction buffer (10 mM Tris HCl; pH 8) containing protease inhibitor cocktail tablet (Complete mini-Roche Diagnostics). Homogenates were then centrifuged at 13000 rpm at 4°C and supernatant was retained. Protein concentration in the supernatant was determined by Coomassie Plus Bradford assay reagent (Pierce, Rockford, IL, USA) using BSA as a standard. Hippocampi from animals with the same genotype (*n* = 7 Tg*hCBS*60.4 and *n* = 8 control) were pooled together to obtain sufficient amount of tissue for processing. CBS activity was analyzed as previously described [36] by employing Biochrom 30 amino acid analyzer to measure cystathionine levels. Briefly, the reaction mixture contained Na-N, N-Bis (2-hydroxyethyl) glycine buffer (200 mM; pH 8.6), DL-Homocysteine (10 mM), L-Serine (5 mM), pyridoxal phosphate (50 µM) and AdoMet (250 µM). The reaction was started by the addition of 30 µg of dialysed protein extract and CBS activity was measured in terms of nmoles of cystathionine formed per milligram of protein per hour at 37°C. In the case of pooled hippocampi, CBS activity was measured in triplicate on the same extracts in two independent experiments.

### Amino acid analysis

Amino acid analysis was done by Biochrom 30 amino acid analyzer as previously described [36]. In summary, 300 µg of undialysed protein extracts prepared for CBS activity measurements were reduced by dithiothreitol and then treated with an equal volume of 10% sulfosalicylic acid followed by centrifugation. The supernatant was then analysed for amino acid content by using the amino acid analyser. Protein concentration in the extracts was determined by Coomassie Plus Bradford assay reagent (Pierce, Rockford, IL, USA). Amino acids were quantified by comparing peak area to a known standard using EZChrom Elite software. Results are expressed as nmoles amino acid/mg protein.

### Electrophysiology

The experiments were conducted with 6–7 month-old mice. Transverse hippocampal slices (400 µm) were obtained as previously described [68] in mice anesthetized with halothane before decapitation. Slices were prepared in ice-cold artificial cerebrospinal fluid (aCSF) and placed in a holding chamber for at least 1 hr. The composition of aCSF was as follows (in mM): NaCl 124, KCl 3.5, MgSO_4_ 1.5, CaCl_2_ 2.3, NaHCO_3_ 26.2, NaH_2_PO_4_ 1.2, and glucose 11, pH 7.4. A single slice was transferred to the recording chamber at a time and continuously superfused with aCSF pre-gassed with 95% O_2_/5% CO_2_. Extracellular recordings were obtained at room temperature from the apical dendritic layer of the CA1 area using micropipettes filled with 2 M NaCl. Presynaptic fiber volleys (PFVs) and field excitatory postsynaptic potentials (fEPSPs) were evoked by electrical stimulation of Schaffer collaterals and commissural fibers located in the *stratum radiatum*. The averaged slope of three PFVs and fEPSPs was measured using the Win LTP software [69]. To evaluate the level of receptor activation, the fEPSP/PFV ratio was plotted against stimulus intensity (from 500 to 900 µA). In order to investigate long-term potentiation (LTP) of synaptic transmission, a test stimulus was applied every 10 sec and adjusted to get a fEPSP with a baseline slope of 0.1 V/sec. The averaged slope of 3 fEPSPs was measured for 15 min before theta-burst stimulation (TBS), consisting of 5 trains of four 100 Hz pulses each, separated by 200 ms and delivered at the test intensity. This sequence was repeated three times with an interburst interval of 10 s. Testing with a single pulse was then resumed for 60 min to determine the level of LTP.

### Behavioral assessment

Behavioral studies were done on 5–6 month-old Tg*hCBS*60.4 and wild-type littermate control male mice. Animals were first submitted to the passive avoidance test, and they were submitted to the rotarod test one week later.

The passive avoidance apparatus (Panlab, Bioseb, France) consisted of two compartments separated by a guillotine door. One compartment was brightly lit; the other compartment was dark with a grid floor connected to an electrical scrambler. The test consisted in an acquisition session and a retention session, conducted 24 h apart. On the acquisition session, the animal was placed in the lit compartment. After 30 s, the guillotine door was opened. As soon as the animal entered the dark compartment, the guillotine door was closed and an electric shock (0.4 mA, 0.5 s) was delivered through the grid-floor. An animal which did not enter into the dark compartment within 300 s after the guillotine door was opened was discarded from the experiment. The retention session was conducted in the same way as the acquisition session except that the shock was not delivered if the animal entered the dark compartment. The latency to enter into the dark compartment was recorded in the acquisition session and in the retention session.

Motor coordination and balancing was tested with an accelerating rotarod (TSE systems; Bad Homburg, Germany). The rotating rod was elevated 10 cm off the floor, had an axis diameter of 3.5 cm and a striated surface made of black rubber. Animals were trained to stay on the rotarod for two 11 min sessions, conducted 24 hours apart. For each session, the animal was placed on the rod which rotates at 1 revolution per min (rpm) for the first min (habituation period). An animal which fell from the rod during the habituation period was placed again on the rod; falls occuring during this period were not taken into account in the analysis of the performance. Then, the speed of the rod progressively accelerated from 1 to 30 rpm (10 min accelerating period). During the accelerating period, animals were allowed to fall three times. They were placed again on the rod after the 1st and 2nd fall, and removed from the apparatus after the 3rd fall. The first, second and third fall latencies (min: 0 s - maximum: 600 s if no falls occurred) were recorded.

### Data analysis

All results are expressed as mean ± S.E.M. For statistical analysis, group means were compared using the Student unpaired two-tailed *t*-test, or the Mann-Whitney-Wilcoxon test when variables were not normally distributed. For electrophysiology experiments, *p*-values were calculated using multivariate analyses of variance followed by post-hoc unpaired *t* tests, in order to take into account the correlations inherent in repeated measures data. Performance on the rotarod was compared using repeated measures ANOVA with falls as repeated measures. In all cases, differences were considered significant when the *p*-value≤0.05.

## Supporting Information

Figure S1 **Coomassie blue staining of the SDS-PAGE blotted in (A)** **fig. 2A and (B**) **fig. 2B** **.**
(TIF)

Table S1 **Limited metabolic profiling of hippocampus.**
(DOC)
