# Supplementary figures and images for: Brain Phenotype of Transgenic Mice Overexpressing Cystathionine β-Synthase
Source: PLoS One. 2012 Jan 12;7(1):e29056. doi: 10.1371/journal.pone.0029056 (PMC3257219; doi:10.1371/journal.pone.0029056)

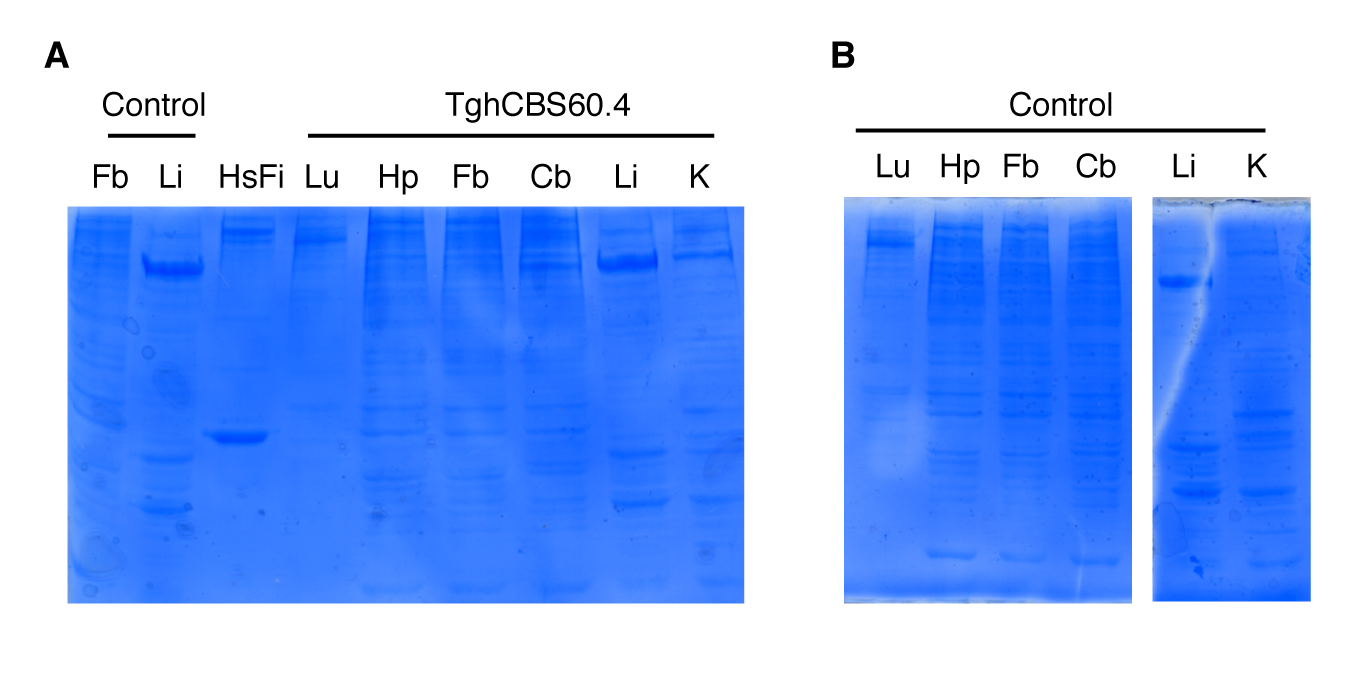

Supplement: Figure S1 — Coomassie blue staining of the SDS-PAGE blotted in (A) fig. 2A and (B ) fig. 2B . (TIF) [file pone.0029056.s001.tif]
