# Supplementary material for: Brain Phenotype of Transgenic Mice Overexpressing Cystathionine β-Synthase
Source: PLoS One. 2012 Jan 12;7(1):e29056. doi: 10.1371/journal.pone.0029056 (PMC3257219; doi:10.1371/journal.pone.0029056)
Supplement: Table S1 — Limited metabolic profiling of hippocampus. (DOC) [file pone.0029056.s002.doc]

|  | Control (n=8) | Tg*hCBS*60.4 (n=7) | Ratio Tg/Control |
| --- | --- | --- | --- |
| Taurine | 240.03 | 259.11 | 1.08 |
| Ser | 24.06 | 26.33 | 1.09 |
| Glu | 198.46 | 227.84 | 1.15 |
| Gly | 24.06 | 24.68 | 1.03 |
| Met | 1.25 | 1.18 | 0.95 |
| Cystathionine | 1.80 | 2.12 | 1.18 |
| Total cysteine | 1.63 | 1.40 | 0.85 |
| Total homocysteine | BD | BD | ND |

Data are expressed in nmoles/mg protein and correspond to values from pools. BD: below detection. ND: not determined.
